# Fifty-Year Trends Reveal Reversal from Recovery to Re-eutrophication and Reinforced Anoxia in a Managed Mountain Lake

**DOI:** 10.1007/s10021-025-01003-5

**Published:** 2025-08-29

**Authors:** Eric Weniger, Ruben Sommaruga

**Affiliations:** Department of Ecology, Universität Innsbruck, Technikerstr. 25, 6020 Innsbruck, Austria

**Keywords:** Climate change, Ecosystem management, Anaerobic mineralization, Phosphorus dynamics, Hypolimnetic withdrawal

## Abstract

Anoxia in lakes has intensified in recent decades, threatening ecosystem functioning. Yet, the mechanisms driving long-term trends in anoxia intensity and duration are complex, especially in managed ecosystems, where field data are limited. Using a 50-year dataset from a lake affected by both eutrophication and restoration measures, we examined annual oxygen dynamics, assessing the effect of external drivers, such as climate warming and hypolimnetic withdrawal effectiveness, and of in-lake processes influencing anoxia. Breakpoint analysis revealed a major ecosystem regime shift around 1996, reversing the earlier recovery trend. Between 1972 and 1996, both the anoxic factor and hypolimnetic total phosphorus concentrations declined, but both rose significantly afterward, with phosphorus concentrations eventually exceeding pre-restoration levels, despite declining watershed inputs. This reversal coincided with a marked increase in thermal stratification duration, which likely intensified deoxygenation by limiting oxygen renewal in the hypolimnion. Our results also show that higher anoxia levels in 1 year significantly reinforced anoxia in the following year, suggesting a self-sustaining feedback mechanism. In addition, our results provide evidence that anaerobic mineralization is important to this feedback, accumulating reduced compounds that further enhance deoxygenation. Despite management efforts, the intensification of internal phosphorus loading and the accumulation of reduced substances have progressively diminished the effectiveness of the cost-effective hypolimnetic withdrawal system implemented since 1970. Our findings demonstrate how the emergence of reinforcing feedbacks, linking oxygen depletion, internal phosphorus release, and climate-driven stratification, can undermine traditional restoration strategies. This highlights the urgent need for adaptive management that explicitly addresses these interacting mechanisms among oxygen dynamics, nutrient cycling, and climate warming.

**Supplementary Information:**

The online version contains supplementary material available at 10.1007/s10021-025-01003-5.

## Highlights

- A breakpoint in 1996 marked a shift from recovery to renewed eutrophication and anoxia.
- Longer thermal stratification periods intensified deoxygenation and internal phosphorus loading.
- Anaerobic mineralization increased despite stable oxygen demand, reinforcing deoxygenation
- Anoxia extent and duration in 1 year positively reinforced trends in subsequent years.
- Hypolimnetic withdrawal lost effectiveness as a restoration tool under changing lake conditions.

## Introduction

Dissolved oxygen is a critical factor in aquatic ecosystems that influences habitat structure, biodiversity, water quality, and greenhouse gas emissions (Encinas Fernández and others 2014; North and others 2014; Rosenberg and others 1991). Globally, aquatic systems are experiencing deoxygenation (Jansen and others 2024; Rose and others 2024), with declines in dissolved oxygen observed in both surface and deep waters of many lakes (Foley and others 2012; Jenny and others 2016; Jane and others 2023; Lewis and others 2024). This trend is particularly evident in the hypolimnion, where oxygen concentrations fluctuate significantly throughout the thermal stratification period (Kiefer and others 2020). In meso- and eutrophic lakes, the oxygen storage capacity of the hypolimnion can be exhausted before the end of the stratification period, resulting in anoxic conditions. Low-oxygen environments render habitats unsuitable for fish and most invertebrates, either due to insufficient oxygen for respiration or increased concentration of toxic by-products from anaerobic mineralization (Wang and Chapman 1999). Further, the presence of anoxic conditions exacerbates the stress from other environmental factors, such as elevated temperatures, further compromising organism survival. As a result, anoxic water layers typically support only anaerobic microorganisms and a few specialized eukaryotic species (Rosenberg and others 1991).

The depletion of dissolved oxygen in the hypolimnion is closely linked to changes in phosphorus dynamics within the lake because it leads to an increase in the availability of this limiting nutrient, which in turn has profound effects on ecosystem functioning (Vollenweider 1968; Nürnberg 1995; Foley and others 2012). Historically, many lakes in central Europe and elsewhere underwent eutrophication in the mid-20 century, largely due to increased agricultural activity and urbanization (Schindler 1977). This process led to a substantial increase in phosphorus levels that can trigger harmful algal blooms, fish mortality, and shifts in community structure (Vollenweider 1968; De Jonge and others 2002; Meerhoff and others 2022). However, the relationship between oxygen depletion and phosphorus dynamics is more complex than once thought (Müller and others 2019; Kiefer and others 2020). A significant challenge in restoring lakes affected by anthropogenic eutrophication is that once phosphorus is introduced, it may not easily leave the system. Over time, phosphorus tends to accumulate in lake sediments (Boström and others 1988; Heinrich and others 2021), which act as both a sink and a source for this element. Through the process of internal loading, phosphorus can be released back into the hypolimnion, further contributing to anoxia (Mortimer 1941; Boström and others 1988; Kowalczewska-Madura and others 2010). This, in turn, compromises the success of reoligotrophication strategies (Matthews and Effler 2006).

Lewis and others (2024) have recently identified a positive feedback mechanism where the decline of dissolved oxygen in the hypolimnion initiates a chain of processes that leads to a worsening of oxygen conditions in the future. The consequence is that when anoxic conditions establish, it increases the extent of anoxia in subsequent years. Specifically, the decline of oxygen in the hypolimnion facilitates the onset of anoxia near the sediment, which promotes internal phosphorus loading and stimulates algal growth. As this excess biomass decomposes, it sinks to the sediment, where the increased mineralization of biomass exacerbates hypolimnetic anoxia, perpetuating the cycle of oxygen depletion and internal loading (Lewis and others 2024). This knowledge derived from an analysis of over 600 lakes and the authors noted the existence of a large variance across lakes. Consequently, the identification of the individual processes involved in this feedback mechanism using long-term data from a single lake has not yet been explored in detail.

Another important mechanism for understanding anoxia dynamics in the water column is the change in concentrations of substances involved in the anaerobic mineralization of organic matter. Under anoxic conditions, microorganisms shift to reducing other terminal electron acceptors (TEAs) to achieve the anaerobic oxidation of organic carbon (Canfield and others 1993). The energy gained from reducing different TEAs follows the so-called ‘redox ladder’, that is from the most energetically favorable (oxygen reduction) to the least one (methanogenesis) (Berner 1981; Stumm and Morgan 2013). As anoxia persists, the progression along the redox ladder continues, facilitating further reductions (Berner 1981). The resulting reduced substances such as methane, manganese, ammonium, soluble iron, and sulfide (Carignan and Lean 1991; Gelda and others 1995; Steinsberger and others 2020) act as negative oxygen equivalents, because when in contact with dissolved oxygen, they become reoxidized (Matzinger and others 2010; Steinsberger and others 2017, 2020).

Our objective in this study was to account for the series of interconnected processes influencing anoxia trends in a lake with a history of eutrophication and restoration measures, including the implementation of hypolimnetic withdrawal (HW). In addition to analyzing long-term trends in oxygen dynamics, we assessed the contribution of different mechanisms such as the duration of thermal stratification and HW efficiency, as well as of internal feedback mechanisms to the observed changes in anoxia. To achieve this, we examined 50 years of data on key physico-chemical parameters, including oxygen, phosphorus, oxygen demand rates, the anoxic factor, TEAs, ammonium, HW effectiveness, and stratification dynamics, using linear regressions and breakpoint analyses, as well as variance partitioning analysis.

## Material and Methods

### Study Site and Basic Measurements

Samples were collected from the Long-Term Socio-Ecological Research (LTSER) site, Lake Piburg, Austria (47° 11′ 42″ N, 10° 53′ 20″ E). This subalpine lake is surrounded by a forest, has an area of 13.4 ha, a maximum depth of 24.6 m, and is situated at 913 m above sea level. The 1% attenuation depth of PAR ranges from ca. 11 to 13 m, so light does not reach the lake bottom. During the early 1960s, Lake Piburg became eutrophic due to the uncontrolled use of fertilizers in its catchment area and the increasing recreational use of the lake (Pechlaner 1980). To mitigate the problem, several countermeasures were implemented to reduce nutrient loading. In 1970, an ‘Olszewski pipe’ for selective HW was installed at the lake’s deepest area, as part of restoration efforts (Pechlaner 1980). The 8.9 cm diameter pipe passively siphons nutrient-rich, oxygen-depleted water from a depth of approximately 23–24 m, discharging it outside the lake’s catchment. The lake’s mountainous location provided an elevational difference of 11 m between the pipe’s inlet and outlet (total length: 639 m), enabling gravity-driven flow without the need for external energy. In 1983, the lake and its surrounding catchment were designated as a landscape conservation area, resulting in stricter regulation of agricultural practices. In addition, recreational use was restricted, including the establishment of a single authorized bathing area equipped with sanitary services.

Since 1972, monthly measurements of key limnological parameters have been recorded at eight depths (0 m, 3 m, 6 m, 9 m, 12 m, 15 m, 18 m, 21 m, and 24 m), with only a few inconsistencies in sampling, primarily due to inaccessibility caused by a thin ice cover. Water temperature has always been measured using a calibrated thermometer (0.1 °C resolution) placed inside a 5-L vertical water sampler. Oxygen concentrations were measured by the Winkler titration method. Chlorophyll-a, used as a proxy for algal biomass, was extracted with alkaline acetone, measured by spectrophotometry and calculated after Lorenzen (1967). Total phosphorus (TP) was determined by spectrophotometry after acid digestion, using the molybdenum blue reaction (Vogler 1966). Sulfate and nitrate were analyzed by spectrophotometry and later, by ion chromatography, while ammonium concentrations were measured using spectrophotometry. If an instrument or method changed during the study period, internal laboratory comparisons were statistically evaluated and since 1980s, quality control measures have included regular participation in ring tests (CNR-IRSA Institute, Italy; and NIVA, Norway) and verification of ion sum balances. An overview on data availability during the 50 years is found in Table S1.

### Stratification Duration

To calculate the duration of the stratification period, monthly temperature profiles were interpolated with a cubic spline function to obtain daily values (Livingstone 2003; Woolway and others 2021). This interpolation creates a smooth curve through the measured data points to estimate intermediate temperatures. Second, the onset and termination times were calculated based on Schmidt stability (S) using the interpolated temperature data. The open-source R package “rLakeAnalyzer” (Winslow and others 2019) was used, which includes the function schmidt.stability. To define the period of stratification or mixed conditions, the critical Schmidt stability (Scrit) was derived by calculating Schmidt stability (S) assuming a uniform temperature of 6 °C in the epi- and metalimnion (0 m–14 m) and a uniform temperature of 4 °C in the hypolimnion (14 m–24 m). For the study lake, this yields a Scrit value of 10.04 J/m^2^ (Niedrist and others 2018). Then, S was calculated for every day throughout the study period. When S was smaller than Scrit, the water column was considered mixed. Conversely, when S exceeded Scrit, the water column was classified as stratified. To determine the duration of stratification for each year, the number of consecutive days with S > Scrit was counted. The more irregular and variable winter stratification period was not considered.

### Terminal Electron Acceptors

To analyze changes in the importance of anaerobic mineralization over time, concentrations of terminal electron acceptors (TEAs) were computed based on volume-weighted sulfate and nitrate concentrations. Weighting factors were applied to these concentrations because their metabolization yields different amounts of energy. The energy yield per mole of oxygen in aerobic respiration served as the reference. Whereas the reduction of 1 mol of oxygen yields -29.98 kJ, that of sulfate yields -5.9 kJ, so sulfate reduction yields 19.7% of the energy of aerobic respiration, resulting in a weighting factor of 0.197 (Stumm and Morgan 2013). Nitrate reduction via denitrification yields -28.4 kJ or -19.6 kJ via nitrate reduction, giving an average weighting factor of 0.84 for nitrate. Sulfate and nitrate concentrations (in mmol/L) were multiplied by their respective weighting factors to determine the total weighted TEA concentrations.

### Anoxic Factor

The anoxic factor (AF) was calculated for each year and for the summer stratification period following the method described by Nürnberg (1995), which is determined using oxygen profiles and lake morphometric information. Anoxia was defined as dissolved oxygen concentrations ≤ 1 mg L^– 1^. The AF integrates the spatial extent and duration of anoxic conditions into a single annual metric expressed in days.

### Concentrations

Concentrations of oxygen, phosphorus, chlorophyll-a, TEA’s, and ammonium were volume-weighted and averaged for each year over the summer stratification period. Total phosphorus concentrations at the single lake inflow (a small and shallow stream) are reported as the annual medians with interquartile ranges.

### Rates

Rates of oxygen, TEA, and ammonium demand/accumulation during summer stratification were calculated using a linear regression model of the respective volume-weighted concentration against days since stratification onset for each year. The resulting slopes represent volumetric demand or accumulation rates. For oxygen, only the first four measurements following the onset of stratification were included to avoid late-season data, when oxygen was already depleted, thereby improving the fit of the regression model. This adjustment was not necessary for TEA’s and ammonium, as their concentration did not exhibit comparable depletion dynamics.

### Contribution of Hypolimnetic Withdrawal (HW)

To evaluate the long-term effect of HW on epilimnetic TP concentrations, we applied an empirical model developed by Nürnberg (2007) and subsequently supported by additional case studies (Nürnberg 2009). The model relates the cumulative areal export of total phosphorus via HW to the proportional reduction in epilimnetic TP concentrations, calculated as:

$${\text{TP}}_{\text{Epilimnion }}\text{reduction} \left(\%\right)=\left(0.471-0.331\times {\text{log}}_{10}\left(\text{cumulative TP Export}\right)\right)\times 100$$

where, *cumulative TP export* is the total mass of TP removed via HW, normalized by lake surface area, and accumulated over consecutive years of operation. To apply the model, we used monthly measurements of TP concentration in the withdrawn hypolimnetic water and the corresponding HW flow rates. Annual TP export was obtained by summing monthly values and normalizing by lake surface area to yield annual areal TP export. The cumulative TP export was calculated as the running total of annual areal TP exports across the entire study period. This cumulative value was then used as input to the model to estimate annual relative reductions in epilimnetic TP concentration. To assess the effectiveness of HW in individual years, we computed year-to-year differences in modeled TP reduction, which represents the annual benefit attributable to on-going phosphorus removal.

To examine whether interannual variation in HW effectiveness influenced lake anoxia or phosphorus dynamics, we calculated year-to-year changes (Δ) in total in-lake phosphorus (TP) stock and in the anoxic factor, and related these to the average HW outflow rate. TP stock was estimated for each sampling date by summing depth-specific phosphorus masses ([TP] × layer volume) across the water column, and then averaging annually. Year-to-year differences in TP stock (ΔTP) and AF (ΔAnoxia) were then regressed against the average annual HW outflow rate to assess whether higher HW volumes corresponded to greater reductions in TP or anoxia. To account for potential delayed effects, we also tested lagged relationships. As a complementary analysis, we assessed the relationship between modeled epilimnetic TP reductions and AF values to further explore the association between HW effectiveness and lake anoxia.

Finally, to quantify the influence of key mechanisms on anoxia, we conducted a multiple linear regression with annual AF as the response variable. Predictors were selected to represent the primary potential mechanistic controls on anoxia: hypolimnetic TP concentration (internal loading), stratification duration (climatic influence), and modeled epilimnetic TP reduction (HW effectiveness). To evaluate the relative importance of each predictor, we applied the Lindeman–Merenda–Gold (LMG) method using the calc.relimp() function from the “relaimpo” package in R (Groemping 2006). This method decomposes the model’s total R^2^ into non-negative shares for each predictor by averaging their contribution across all possible orderings, thereby providing an unbiased estimate of individual variable importance.

### Breakpoint Analysis and Other Statistical Tests

A breakpoint analysis was conducted to detect potential shifts in trends over time using the open-source program "R" and the "segmented" package (Muggeo 2008, R Core Team 2023). For each breakpoint suggested, the following steps were taken to confirm or reject it. First, a linear regression was applied to all measurements, with time as the predictive variable. Then, a Davies-Test (Davies 1987, 2002) was used to test whether the slopes before and after a suggested breakpoint were significantly different. If the slopes differed significantly, the breakpoint was accepted. As a second validation, the Akaike Information Criterion (AIC) was used to compare model performance. A linear model without breakpoints was compared with one incorporating the suggested breakpoint, and the model with the lower AIC was considered as the best. If the Davies-test did not support a breakpoint, but the AIC suggested improved model performance, the breakpoint model was used.

For all models, diagnostic functions from the "DHARMa" package (Hartig 2022) were used to confirm that no assumptions were violated. A non-parametric bootstrap approach was used to test whether a slope was significantly different from zero. This was done by reshuffling all data points, recalculating the slope for each reshuffled dataset (repeated 10,000 times), and comparing the original slope with the distribution of reshuffled slopes. If the original slope was greater than 5% of the reshuffled slopes (*p* < 0.05), the null hypothesis was rejected, indicating the slope was significantly different from zero.

Correlations between annual averages of parameters, known to have causal relationships, were tested using Pearson’s product-moment correlation in R. To assess whether conditions in 1 year influenced those in the following year, independent of long-term trends, we first removed temporal trends from the time series of both the anoxic factor and hypolimnetic phosphorus concentrations by regressing each against time and extracting the residuals. Then, we calculated the lag-1 Pearson's correlation coefficient between residuals at year *t* and *t* + *1* to quantify the degree of year-to-year dependence.

## Results

### Anoxia and Phosphorus Dynamics

**Figure 1:**
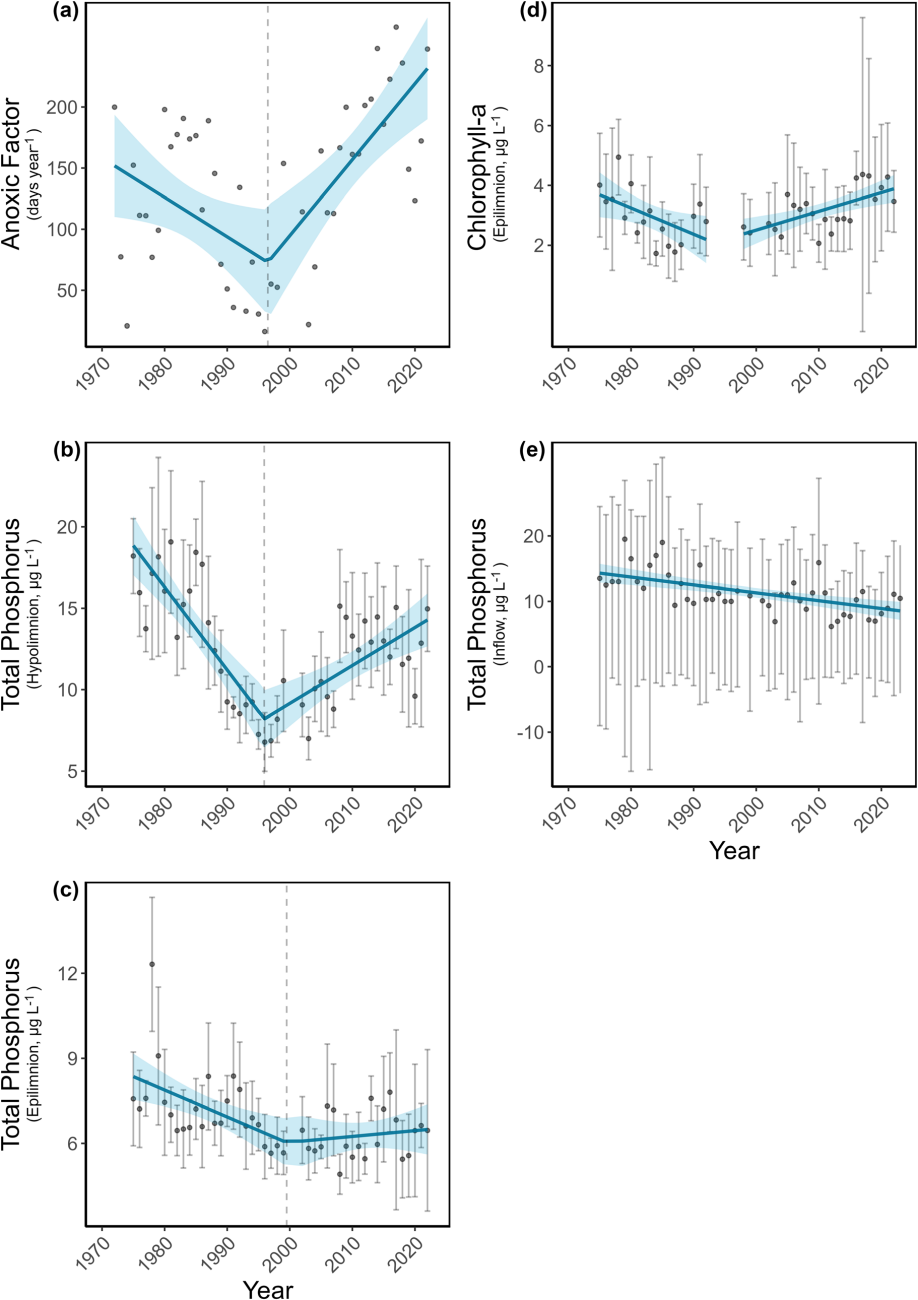
Temporal trends for the anoxic factor (**a**), hypolimnetic phosphorus (**b**), epilimnetic phosphorus (**c**), epilimnetic chlorophyll-a concentrations (**d**), and TP concentrations in the inflow (**e**). Continuous lines show linear regression models with year as the predictor and the respective parameter as the response variable. Shaded areas indicate the 95% confidence intervals of these regressions. Data points with error bars represent annual means during the stratification period, with error bars indicating the standard error of the mean. In panel (**e**), data points represent the annual median, and error bars show the interquartile range.

A breakpoint analysis identified a shift in the trend of the anoxic factor in 1996 (Figure 1a, Davies-Test, *p* < 0.01). From 1972 to 1996, the AF showed a decrease at a rate of −3.22 d year^– 1^ which was not statistically significant (non-parametric bootstrap, 95% CI [−6.78, −0.01], *p* > 0.05). By contrast, from 1997 to 2022, the AF increased significantly at a rate of 6.22 d summer^– 1^ year^– 1^ (non-parametric bootstrap, 95% CI [2.66, 9.58], *p* < 0.001), from 76 d year^– 1^ to 232 d year^– 1^. The AF reached its initial value in 2009 and continued to surpass it thereafter. After removing the long-term trend, the AF residuals had a significant positive lag-1 correlation (*R* = 0.37, *p* < 0.01, Table S2).

The trend for volume-weighted hypolimnetic total phosphorus concentration showed also a breakpoint in 1996 (SE = 1.75, *p* < 0.001, Figure 1b). From 1975 to 1996, concentrations decreased at − 0.5 µg L^– 1^ year^– 1^ (non-parametric bootstrap, 95% CI [-0.75, -0.25], *p* < 0.001). However, from 1996 to 2022, they increased at 0.23 µg L^– 1^ year^– 1^ (non-parametric bootstrap, 95% CI [0.1, 0.37], *p* < 0.001). The phosphorus concentration in the hypolimnion was highly significantly correlated with the anoxic factor (*R* = 0.57, *p* < 0.001, Table S2) and its lag-1 residuals also showed a highly significant correlation (*R* = 0.42, *p* < 0.001, Table S2).

A breakpoint for the trend in volume-weighted epilimnetic TP concentration was found in 2000 (Figure 1c). Although this breakpoint was not statistically significant (Davies-Test, *p* > 0.05), the AIC difference between the simpler model without a breakpoint and the breakpoint model was 2.34. This AIC difference provides moderate support for selecting the breakpoint model for further analysis. From 1975 to 2000, epilimnetic phosphorus concentrations declined at rate of -0.1 µg L^– 1^ year^– 1^ (non-parametric bootstrap, 95% CI [-0.16, -0.02], *p* < 0.05). After the breakpoint, no further reduction in epilimnetic phosphorus concentrations was detected and levels remained stable at around 6.3 µg L^– 1^ (non-parametric bootstrap, 95% CI [− 0.04, 0.07], *p* > 0.05). A significant correlation between hypolimnetic and epilimnetic TP concentrations was found (*R* = 0.30, *p* < 0.05, Table S2). No significant trend was observed in the epilimnetic N:P ratio (non-parametric bootstrap, *p* > 0.05, Figure S1). In contrast, the hypolimnetic N:P ratio significantly declined (non-parametric bootstrap, *p* < 0.05).

For chlorophyll-a, a breakpoint was identified in 1989 (Davies-Test, *p* < 0.001, Figure 1d); however, this result is uncertain due to the data gap between 1992 and 1997 (Table S1). Therefore, two separate linear models were constructed and analyzed individually for the periods before and after this gap. From 1975 to 1992, epilimnetic chlorophyll-a concentrations significantly decreased at a rate of − 0.09 µg L^– 1^ year^– 1^ (non-parametric bootstrap, 95% CI [− 0.16, − 0.01], *p* < 0.05). From 1998 to 2022, epilimnetic chlorophyll-a concentrations increased significantly at a rate of 0.06 µg L^– 1^ year^– 1^ (non-parametric bootstrap, 95% CI [0.02, 0.10], *p* > 0.01). Epilimnetic chlorophyll-a concentrations were significantly positively correlated with epilimnetic TP levels (R = 0.32, *p* < 0.05, Table S2), despite a gradual long-term decline in inflow TP concentrations (non-parametric bootstrap, slope = -0.12, 95% CI [− 0.18, − 0.06],* p* < 0.001, Figure 1e). The trend for inflow TP did not show a significant breakpoint (Davies-Test, *p* > 0.05), nor was it significantly correlated with the AF (R = -0.31, *p* > 0.05).

### Mineralization Dynamics

**Figure 2:**
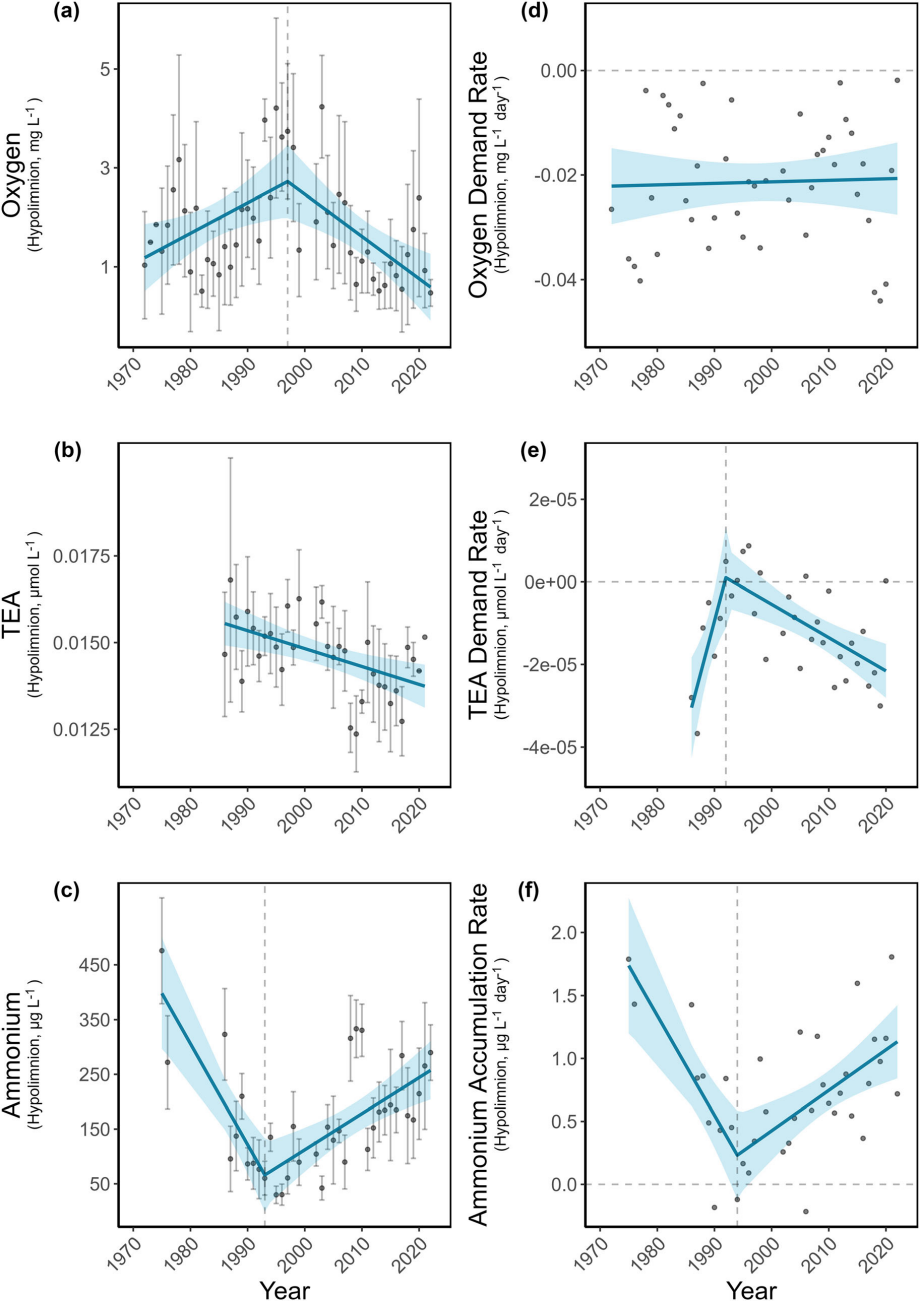
Temporal trends for the weighted hypolimnetic oxygen concentration (**a**), weighted TEA concentrations (**b**), weighted ammonium concentrations (**c**), oxygen demand rate (**d**), TEA demand rate (**e**), and ammonium accumulation rate (**f**). Continuous lines represent regression models with time as predictor and the respective parameter as response variable. The shaded area represents the 95% confidence interval of that regression. Data points represent the mean during stratification period. Error bars represent the standard error of the mean.

The hypolimnetic oxygen concentrations showed a breakpoint in 1997 (Davies-Test, *p* < 0.01, Figure 2a) and until this year, values increased at a rate of 0.06 mg L^– 1^ year^– 1^ (non-parametric bootstrap, 95% CI [0.01, 0.11], *p* < 0.05). After the breakpoint, oxygen levels dropped significantly at a rate of -0.09 mg L^– 1^ year^– 1^ (non-parametric bootstrap, 95% CI [-0.14, -0.03], *p* < 0.01). The oxygen demand rate showed no significant trend throughout the entire study period (non-parametric bootstrap, slope = 2.9 * 10^–5^, 95% CI [-0.001, 0.001], *p* > 0.05, Figure 2d).

TEA concentrations showed a continuous decline from 1986 to 2022 (non-parametric bootstrap, slope = -5.18 × 10^−5^, 95% CI [-8.56 × 10^−5^, -1.73 × 10^−5^], *p* < 0.05, Figure 2b). No breakpoint was statistically supported (Davies-Test, *p* > 0.05) and the best model was linear without a breakpoint. TEA concentrations correlated significantly with the AF (R = -0.48, *p* < 0.05). The TEA demand rate showed a breakpoint in 1992 (Davies-Test, *p* < 0.01, Figure 2e). Before 1992, the TEA demand rate increased significantly at a rate of 5.25 × 10^−6^ mmol L^– 1^ day ^−1^ year^– 1^ (non-parametric bootstrap, *p* < 0.05), but after the breakpoint, demand rates significantly decreased over time at a rate of -8.07 × 10^−7^ mmol L^– 1^ day ^−1^ year^– 1^ (non-parametric bootstrap, *p* < 0.001).

The trend for ammonium concentration in the hypolimnion showed a breakpoint in 1993, which was statistically supported (Davies-Test, *p* < 0.001, Figure 2c). Before the breakpoint, ammonium concentrations significantly decreased at a rate of -18.4 µg L^−1^ day^−1^ year^−1^ (non-parametric bootstrap, 95% CI [-32.73, -6.39], *p* < 0.01), but afterward, they increased at a rate of 6.6 µg L^−1^ day^−1^ year^−1^ (non-parametric bootstrap, 95% CI [2.87, 10.31], *p* < 0.001). Similarly, ammonium accumulation rates had a breakpoint in 1994 (Davies-Test, *p* < 0.001, Figure 2f) and until that year, they decreased significantly at a rate of -0.08 µg L^−1^ day^−1^ year^−1^ (non-parametric bootstrap, 95% CI [-0.14, -0.02], *p* < 0.01), but afterward, they increased significantly at a rate of 0.03 µg L^−1^ day^−1^ year^−1^ (non-parametric bootstrap, 95% CI [0.01, 0.03], *p* < 0.01).

### Duration of Stratification and Role of Hypolimnetic Withdrawal

**Figure 3:**
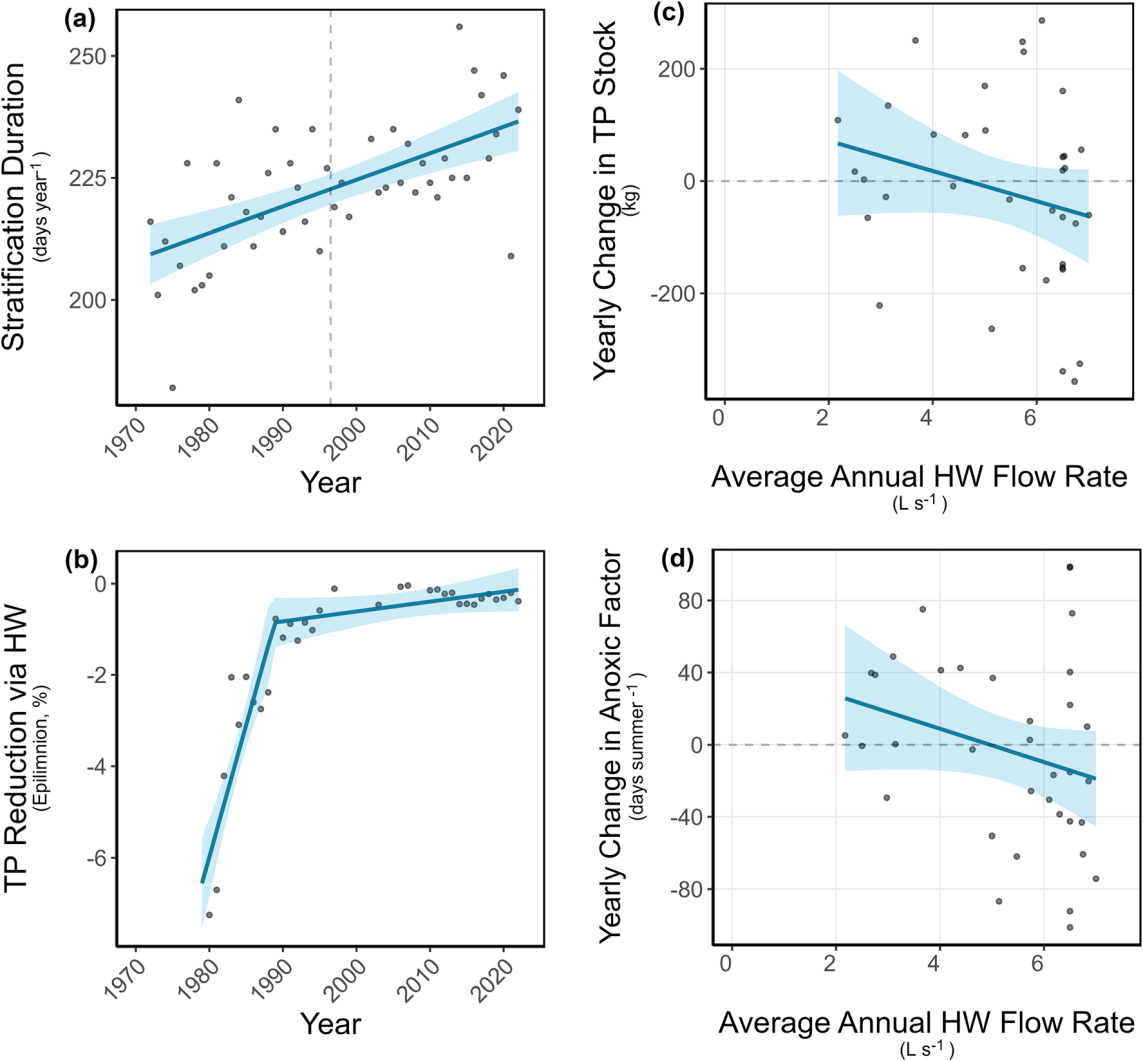
Temporal trends for the stratification duration (**a**), predicted reduction in epilimnetic TP via hypolimnetic withdrawal, HW (**b**) and the relationships between average HW flow rate and year-to-year changes in in-lake TP stock (**c**) and anoxic factor (**d**). Continuous lines represent regression models, and the shaded area represents the 95% confidence interval of that regression.

The duration of the thermal stratification period increased linearly (no breakpoint was statistically supported, Davies-Test, *p* > 0.05) by 0.54 days per year from 1972 to 2022 (non-parametric bootstrap, 95% CI [0.28, 0.78], *p* < 0.001, Figure 3a). By 2022, the stratification duration increased by 28 days compared with 1972. Stratification duration correlated significantly with the AF (R = 0.32, *p* < 0.05, Table S2).

The modeled reduction in epilimnetic TP content showed a breakpoint in 1989 (Davies-Test, *p* < 0.05, Figure 3b). In 1979, the annual reduction of epilimnetic TP by HW was predicted to be around − 6.5%. It significantly declined until it reached about -1% reduction in 1989 (non-parametric bootstrap, slope = 0.58, 95% CI [0.16, 0.97], *p* < 0.05). After that, the annual estimated TP reduction declined significantly at a slower rate, staying consistently below -1% reduction until reaching − 0.13% in 2022 (non-parametric bootstrap, slope = 0.02, 95% CI [0.01, 0.03], *p* < 0.05). No significant correlation was found between the modeled annual TP reduction via HW and the AF (Table S2).

No significant relationship was found between the annual average HW flow rate and the year-to-year change in in-lake TP stock (non-parametric bootstrap, slope = –26.93, 95% CI [–66.85, 11.3], *p* > 0.05; Figure 3c). Similarly, the annual average HW flow rate did not significantly influence year-to-year changes in the AF (slope = –9.26, 95% CI [–20.35, 1.56], *p* > 0.05; Figure 3d). The same analysis was conducted with the HW flow rate and the 1-year lagged changes in anoxia and lake stock TP, but again no significant relationships were found (anoxia: non-parametric bootstrap, slope = -0.77, 95% CI [-12.29, 9.88]), *p* > 0.05; TP: non-parametric bootstrap, slope = -22.6, 95% CI [-56.79, 14.51], *p* > 0.05).

### Relative Importance of Main Drivers

The multiple regression analysis explained 61% of the variance in the AF (*R*^2^ = 0.61; adjusted *R*^2^ = 0.57). Among the predictors, hypolimnetic TP concentration merged as the strongest and only statistically significant variable (*β* = 17.02 ± 3.06 SE, *p* < 0.001). Stratification duration showed a marginal, non-significant effect (*β* = 1.39 ± 0.74 SE, *p* = 0.070), while the modeled epilimnetic TP reduction due to HW was not statistically significant (*β* = 8.97 ± 5.97 SE, *p* = 0.144). Analysis of the relative contribution of each predictor to the model’s explanatory power, based on the Lindeman–Merenda–Gold (LMG) method, showed that hypolimnetic TP concentration accounted for ca. 76% of the model’s *R*^2^, followed by stratification duration (ca. 17%) and HW effectiveness (ca. 7%).

## Discussion

Like other temperate lakes, anoxic conditions in Lake Piburg have increased over time, as indicated by the increase in the anoxic factor (Figure 1a). However, this lake underwent a prolonged period of clear reoligotrophication, during which anoxia intensity and duration improved. Thus, the anoxic factor improved from 1972 to 1997 following hypolimnetic withdrawal and other eutrophication countermeasures, but anoxia has more than doubled in spatial and temporal extent since then (Figure 1a). Consequently, the reversal of the anoxia trend in Lake Piburg poses a significant threat to overall ecosystem integrity and reflects a broader pattern of documented deoxygenation in temperate lakes in recent years (Jane and others 2021, 2023; Lewis and others 2024), a particularly concerning trend for a managed ecosystem.

Low-oxygen concentrations in the water column influence nutrient availability and vice versa. The strongest effect of hypolimnion deoxygenation is the well-established link between anoxia and rising hypolimnetic phosphorus levels due to the increase in phosphorus flux from the sediments (Mortimer 1941; Orihel and others 2017; Gibbons and Bridgeman 2020). This relationship is most pronounced when a threshold of 1.8 mg L^– 1^ dissolved oxygen is crossed (Nürnberg 1995), below which phosphorus release increases significantly, while further oxygen increments above that threshold do not proportionally reduce phosphorus levels (Wang and others 2008). In our study, the anoxic factor and phosphorus concentrations in the hypolimnion were strongly correlated and both had a clear breakpoint in 1996. Before the breakpoint, both parameters declined significantly, but increased thereafter, supporting their causal relationship (Figure 1a and b). However, this correlation does not necessarily always imply cause-effect, because anoxia can be the result of eutrophication following higher P release or the cause of P release (Hupfer and Lewandowski 2008). Although phosphorus release from the sediment was not measured, historical restoration efforts and the long-term decline in phosphorus in the inflow (Figure 1e) suggest that recent increases in in-lake phosphorus and the clear shift in N:P ratio found in the hypolimnion (Figure S1) are primarily due to internal loading (Figure 1b).

The lake was highly eutrophic in the 1960s (Pechlaner 1980), but declines in agricultural activity subsequently reduced external phosphorus loading (Figure 1e). Further, a hypolimnetic withdrawal system was installed to enhance deep-water oxygen conditions and to reduce both phosphorus and reduced substances in the hypolimnion. However, the system’s effectiveness declined over time, as reflected by the modeled reductions in epilimnetic total phosphorus that were most pronounced between 1979 and 1989 and although they continued thereafter at a slower rate (always below –1% annually, Figure 3b), the system’s performance steadily waned. Biofouling, sediment accumulation, and pipe deformation caused by falling trees have likely reduced the pipe’s internal diameter and diminished its outflow capacity. Despite the early benefits of the hypolimnetic withdrawal system, evident in the initial recovery phase of Lake Piburg, there is no evidence over the 50-year observation period of a sustained mass-balance effect on anoxia or phosphorus trends. Modeled reductions in total phosphorus showed no significant correlation with the anoxic factor, nor did hypolimnetic outflow rates correlate with interannual changes in lake phosphorus stock or anoxia extent (Figure 3c and d, Table S2). Although the breakpoint in modeled phosphorus reduction occurred approximately 7 years in advance of that for the anoxic factor and hypolimnetic phosphorus, lag analyses failed to detect a short-term delayed response to withdrawal. These results align with earlier findings in Lake Piburg that measurable improvements in total phosphorus concentrations and phytoplankton biomass following hypolimnetic withdrawal implementation took nearly two decades to become detectable (Pipp and Rott 1995; Tolotti and Thies 2002).

The results from the LMG-based variance partitioning analysis further indicates that withdrawal effectiveness explained only a small fraction of the variance in the anoxic factor. This reinforces the conclusion that other drivers, most notably intensified internal phosphorus loading and prolonged stratification, have exerted stronger, overriding influences on the extent and duration of anoxia.

Transport mechanisms such as seasonal turnover, internal seiche activity, molecular diffusion, and biotic transport can move phosphorus from the hypolimnion to the epilimnion (Carpenter and others 1992; Kamarainen and others 2009; Nürnberg 2009; Haupt and others 2010) with direct implications for phytoplankton dynamics. In Lake Piburg, the significant positive correlation between hypolimnetic and epilimnetic phosphorus concentrations suggests vertical transport, which likely contributed to increased phytoplankton biomass as indicated by a significant positive correlation between epilimnetic phosphorus and chlorophyll-a concentrations (Table S2). During the initial restoration phase (1972–1989), phytoplankton biomass (direct measurements) declined in parallel with reductions in phosphorus concentrations as reported earlier (Tolotti and Thies 2002). In subsequent years, as phosphorus concentrations rose, chlorophyll-*a* levels similarly increased, consistent with the well-established link between phosphorus availability and algal biomass production (Lewis and others 2024). Higher lake productivity is often associated with increased oxygen demand, driven by enhanced biomass production and subsequent organic matter decomposition (Misra et al. 2011). However, our findings do not support this relationship in Lake Piburg, as we did not detect a significant long-term trend in oxygen demand rate (Figure 2d). One possible explanation is the consistently low-oxygen availability in the hypolimnion throughout the study period. In fact, the volume-weighted annual average oxygen concentration in the hypolimnion remained generally below 2 mg L^−1^, with only a few years in the mid-1990s exceeding 3 mg L^−1^ (Figure 2a). Such persistently low-oxygen levels may obscure trends in oxygen demand and promote a shift in microbial respiration toward use of alternative terminal electron acceptors (TEAs), following the redox ladder. Evidence for this shift includes the long-term decline in TEA concentrations based on nitrate and sulfate (Figure 2b) and the concurrent acceleration in TEA demand rates (Figure 2e), both of which suggest intensifying anaerobic mineralization processes. The increasing trend in ammonium accumulation rates further supports this interpretation, which exhibited a distinct breakpoint around 1994 (Figure 2f), coinciding with other critical transitions observed in the lake. As decomposition intensifies, ammonium accumulates more rapidly through microbial mineralization and anoxia favors also its release from the sediment. The parallel increase in ammonium concentrations and accumulation rates, together with the accelerated TEA demand and declining TEA availability, collectively indicate a growing dominance of anaerobic decomposition. This shift enhances the production of reduced compounds such as ammonium and sulfide (here not quantified), which consume oxygen upon re-oxidation, thereby exacerbating hypolimnetic oxygen depletion and expanding the extent of anoxia.

Another important driver to explain the reversal in anoxia trends in Lake Piburg is the fact that since the onset of monitoring, the duration of thermal stratification has increased by ca. 28 days, following an almost linear trend (Figure 3a). Prolonged and stronger stratification in this lake inhibits vertical mixing (Niedrist and others 2018), isolating the hypolimnion from atmospheric oxygen inputs and thereby extending the period of anoxic conditions. The significant correlation between stratification duration and anoxic factor, along with the results from the LMG-based variance partitioning analysis, indicates the influence of this driver on the progression of anoxia, particularly, when considering the period after the breakpoint in the anoxic factor. In addition, earlier onset of stratification shortens the spring mixing period (Niedrist and others 2008), further limiting oxygen replenishment to deep-water layers. As stratification duration lengthens and the ice cover period shortens (Niedrist and others 2018), hypolimnetic oxygen concentrations are expected to decline further, leading to an expansion of the lake volume affected by anoxia (Foley and others 2012; Oleksy and Richardson 2021; Woolway and others 2021). This trend is particularly concerning given Lake Piburgs’s susceptibility to meromixis (Nürnberg 2007).

Based on the above discussion, we propose that the sequence of processes whereby anoxia promotes increased internal phosphorus loading, subsequently fueling eutrophication and enhancing biomass decomposition, which in turn exacerbates anoxia, constitutes a self-reinforcing set of feedback mechanisms. This interpretation is supported by significant correlations between the anoxic factor and all metrics associated with anaerobic mineralization (Table S2) and by the significant lag-1 autocorrelation of detrended residuals of the anoxic factor, which reveal that elevated anoxia in 1 year is significantly associated with increased anoxia in the subsequent year (Table S2). A similar detrended analysis using hypolimnetic volume-weighted TP also showed a significant positive correlation, reinforcing the role of internal loading in sustaining the feedback mechanisms. Finally, extended stratification appears to prolong anoxia, intensifying these processes by providing more time for internal phosphorus loading and organic matter mineralization. Stronger thermal stability and higher temperatures are also known to affect interannual phytoplankton variability in Lake Piburg (Tolotti et al. 2012).

## Conclusions and implications

Our long-term trend analyses provide support for the feedback mechanism driven by internal loading proposed by Lewis and others (2024) and highlight the importance of recognizing the contribution of anaerobic mineralization to anoxia trends. This process, triggered by hypolimnetic deoxygenation, reinforces anoxia temporal trends although at different temporal scales (inter- vs. intra-annual).

Given the worsening oxygen dynamics in the last decades, coupled with the increasing stratification duration, reversing these trends is a challenge. The first step in any lake restoration effort is to minimize external phosphorus inputs, usually the most cost-effective approach (Marsden 1989; Schindler and others 2016). In Lake Piburg, combined inflow phosphorus reduction and the installation of a hypolimnetic withdrawal system successfully shifted conditions from eutrophic to mesotrophic between 1972 and mid-1990s. However, since mid-1990s, accelerating climate warming and increased internal loading rendered these measures insufficient. Although increasing the withdrawal outflow rate beyond current levels may eventually bolster lake restoration, any such benefit will likely be delayed by many years. Therefore, consideration of more technically advanced restoration strategies, such as artificial oxygenation or hypolimnetic mixing, might support the recovery of the lake (Gantzner and others 2009). However, artificial oxygenation may be ineffective in this system, as the withdrawal system already removes more iron than it receives, potentially limiting phosphorus co-precipitation (Psenner 1988). Artificial mixing (Cooke and others 2016; Bryant and others 2024) also poses risks, such as the increase of hypolimnetic temperatures and habitat degradation for cold-water fish species (Beutel and Horne 1999). Nevertheless, with projected rises in air temperature and heatwaves that accelerate deoxygenation (Zhang and others 2025), the described feedback mechanism will very likely exacerbate deoxygenation of an even larger critical volume in the lake with negative consequences for the ecosystem. A clear indication of this is the recent novel record in Lake Piburg of the N-fixer cyanobacterium *Dolichospermum lemmermannii*. Thus, future management strategies must account for these accelerating climate-driven trends to mitigate further ecological deterioration resulting from past and on-going eutrophication (Meerhoff and others 2022).

## Supplementary Information

Below is the link to the electronic supplementary material.

Supplementary file1 (DOCX 86 kb).

## Data Availability

Data used in this study are available from Zenodo DOI: https://zenodo.org/records/15076633
