# Supplementary material for: Fifty-Year Trends Reveal Reversal from Recovery to Re-eutrophication and Reinforced Anoxia in a Managed Mountain Lake
Source: Ecosystems. 2025 Aug 29;28(5):56. doi: 10.1007/s10021-025-01003-5 (PMC12397146; doi:10.1007/s10021-025-01003-5)

Supplementary information

**Fifty-year trends reveal reversal from recovery to re-eutrophication and reinforced anoxia in a managed mountain lake**

Eric Weniger ORCID [0000-0002-4016-7345](https://orcid.org/0000-0002-4016-7345), Ruben Sommaruga* ORCID [0000-0002-1055-2461](https://orcid.org/0000-0002-1055-2461)

Content

Table S1

Table S2

Figure S1

**Corresponding author*; *e-mail*: [ruben.sommaruga@uibk.ac.at](mailto:ruben.sommaruga@uibk.ac.at)

**Table S1.** Data availability of parameters used in the statistical analysis.

| **Parameter** | **Sampling Period** | **Excluded Years** |
| --- | --- | --- |
| Water Temperature | 1972–2022 | 2000 |
| Oxygen | 1972–2022 | 2000–2001 |
| Total Phosphorus | 1975–2022 | 2000–2001 |
| Total Phosphorus – Inflow | 1975–2022 | 2000 |
| Chlorophyll-a | 1975–2022 | 1989, 1992–1997, 2000–2001 |
| TEA’s | 1986–2021 | 2000–2001 |
| Ammonium | 1986–2022 | 2000–2001 |
| DN:DP | 1998–2022 | 2000–2001 |
| Olszewski Outflow | 1980–2022 | 1996, 1998–2002, 2004–2005, 2008–2009 |

**Table S2.** Summary of Pearson correlations and autocorrelations tested in the study. Significant values are labelled in bold.

Processes R *p*-value

| **Anoxia and phosphorus dynamics**  Anoxic Factor ∼ TP_Hypolimnion_ | **0.57** | 𝒑 | < **0**.**001 ***** |
| --- | --- | --- | --- |
| TPHypolimnion ∼ TPEpilimnion | **0.30** | 𝒑 | < **0**.**05 *** |
| TPEpilimnion ∼ Chlorophyll-aEpilimnion | **0.32** | 𝒑 | < **0**.**05 *** |
| **Mineralization dynamics** |  |  |  |
| Chlorophyll-aEpilimnion ∼ OxygenHypolimnion | 0.03 | 𝑝 | > 0.05 |
| Chlorophyll-a_Epilimnion_ ∼ Oxygen demand rate | **-0.33** | 𝒑 | < **0**.**05 *** |
| Chlorophyll-a_Epilimnion_ ∼ TEA | -0.35 | 𝑝 | > 0.05 |
| Chlorophyll-a_Epilimnion_ ∼ TEA demand rate | 0.02 | 𝑝 | > 0.05 |
| Chlorophyll-a_Epilimnion_ ∼ Ammonium | 0.30 | 𝑝 | > 0.05 |
| Chlorophyll-a_Epilimnion_ ∼ Ammonium accum. rate | 0.25 | 𝑝 | > 0.05 |
| Anoxic Factor ∼ Oxygen demand rate | 0.22 | 𝑝 | > 0.05 |
| Anoxic Factor ∼ TEA | **-0.48** | 𝒑 | < **0**.**01 **** |
| Anoxic Factor ∼ TEA demand rate | **-0.61** | 𝒑 | < **0**.**001 ***** |
| Anoxic Factor ∼ Ammonium | **0.51** | 𝒑 | < **0**.**01 **** |
| Anoxic Factor ∼ Ammonium accumulation rate | **0.43** | 𝒑 | < **0**.**01 **** |
| **External Factors**  Anoxic Factor ∼ Stratification duration | 0.31 | 𝒑 | < **0**.**05 *** |
| Anoxic Factor ∼ TP_Inflow_ | -0.21 | 𝑝 | > 0.05 |
| TPEpilimnion ∼ TPInflow | 0.35 | 𝑝 | > 0.05 |
| Anoxic Factor ∼ Modeled TP_Epilimnion_ Reduction | -0.13 | 𝑝 | > 0.05 |
| **Detrended Lagged Correlation (1-year lag)**  Anoxic Factor | **0.37** | 𝒑 | < **0**.**01 **** |
| TPHypolimnion | **0.42** | 𝒑 | < **0**.**001 ***** |

**Figure S1.** Temporal trends in epilimnetic and hypolimnetic dissolved N:P ratios. The X-axis represents years, and the Y-axis displays the annual average ratio. Solid lines indicate linear regression fits with 95% confidence intervals, and error bars denote ±1 standard deviation.


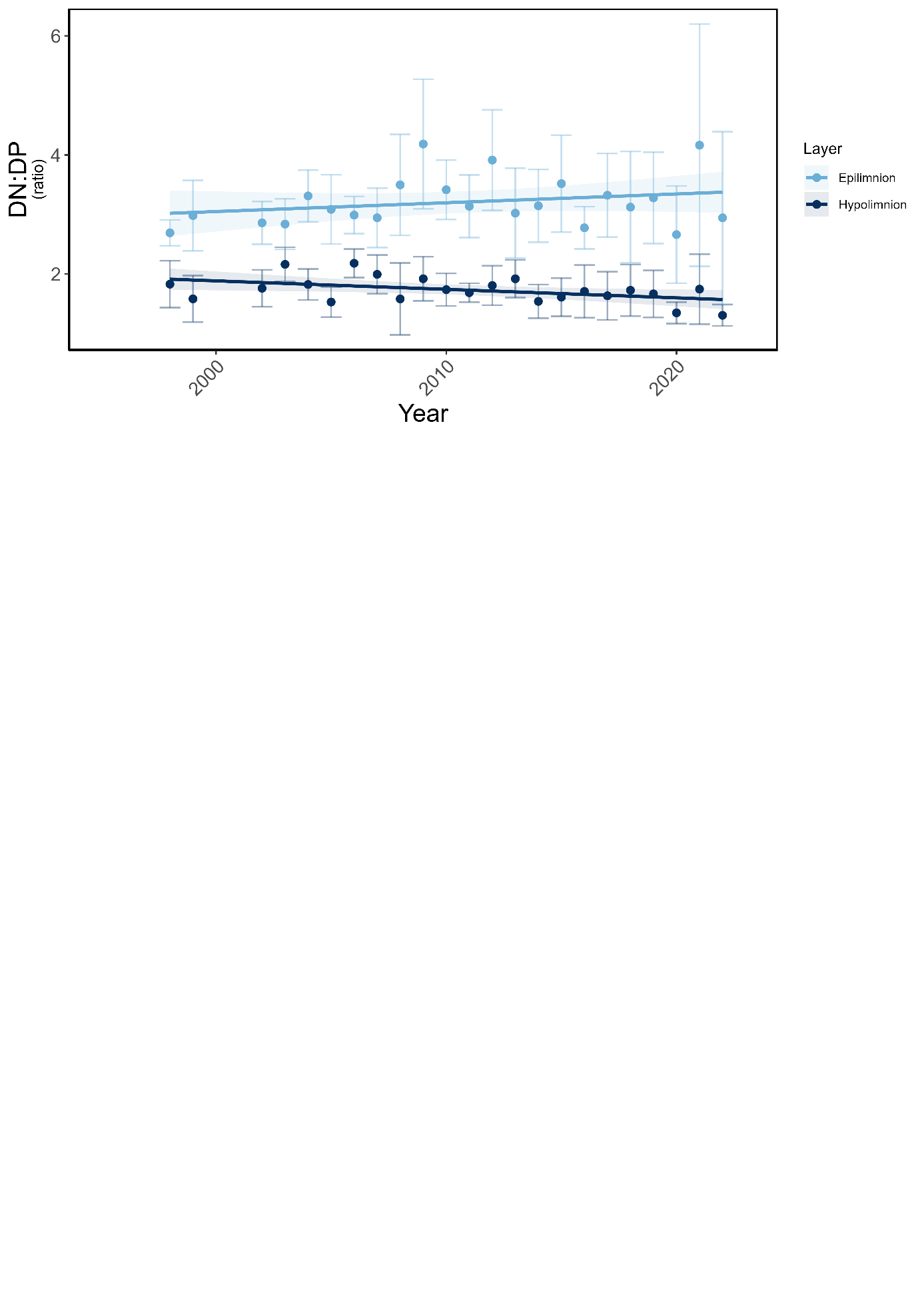

Supplement: Supplementary file 1 — Supplementary file1 (DOCX 86 kb). [file 10021_2025_1003_MOESM1_ESM.docx]
